# Alcohol and substance use among first-year students at the University of Nairobi, Kenya: Prevalence and patterns

**DOI:** 10.1371/journal.pone.0238170

**Published:** 2020-08-28

**Authors:** Catherine Mawia Musyoka, Anne Mbwayo, Dennis Donovan, Muthoni Mathai

**Affiliations:** 1 Department of Psychiatry, College of Health Sciences, University of Nairobi, Nairobi, Kenya; 2 Department of Psychiatry & Behavioural Sciences, Alcohol & Drug Abuse Institute, University of Washington School of Medicine, Seattle, WA, United States of America; University of the Witwatersrand, SOUTH AFRICA

## Abstract

**Objective:**

Increase in alcohol and substance use among college students is a global public health concern. It is associated with the risk of alcohol and substance use disorders to the individual concerned and public health problems to their family and society. Among students there is also the risk of poor academic performance, taking longer to complete their studies or dropping out of university.

This study determined the prevalence and patterns of alcohol and substance use of students at the entry to the university.

**Method:**

A total of 406 (50.7% male) students were interviewed using the Assessment of Smoking and Substance Involvement Test (ASSIST) and the Alcohol Use Disorder Identification Tool (AUDIT). Bivariate logistic regression analyses were used to examine associations between substance use and students' socio-demographic characteristics. Multivariate logistic regression analysis was conducted to examine the predictors of the lifetime and current alcohol and substance use.

**Results:**

Lifetime and current alcohol and substance use prevalence were 103 (25%) and 83 (20%) respectively. Currently frequently used substances were alcohol 69 (22%), cannabis 33 (8%) and tobacco 28 (7%). Poly-substance use was reported by 48 (13%) respondents, the main combinations being cannabis, tobacco, and alcohol. Students living in private hostels were four times more likely to be current substance users compared with those living on campus (OR = 4.7, 95% CI: 2.0, 10.9).

**Conclusion:**

A quarter of the study respondents consumed alcohol and/or substances at the entry to university pushing the case for early intervention strategies to delay initiation of alcohol and substance use and to reduce the associated harmful consequences.

## Introduction

Alcohol and substance use has continued to rise globally, more so among college students [1]. Statistics show a consistency of alcohol and substance use across countries. Globally, a total population of about 275 million people used a psychoactive substance at least once in 2016 [1]. In the United States of America (USA) the rate of substance is rising among those aged 18 to 25 years, with many of them being new users [2]. Alcohol, cannabis, and opioids are the most used substances by those aged 18 to 25 years in America [3]. In this age group, the daily use of marijuana was reported by 2.6 million users, while 3.4 million (10%) had alcohol use disorders [2]. In Europe an estimated 19.1 million young adults (aged 15–34) used substances in 2018 [4]; males used substances twice as much as females with cannabis being the most used substance [4].

In Africa studies conducted in universities in Nigeria, Uganda, Ethiopia and South Africa have found that the prevalence of alcohol and substance use ranged between 27.5% and 62%[5,6]. The prevalence of substance use among undergraduate students in one university in Nigeria was reported at 27.5% [7]. The United Nations Office on Drugs and Crimes (UNODC) 2018 report on substance use in Nigeria, puts the overall past-year prevalence at 14.3 million (14.4%) [1]. While use is reported across all age groups, the highest use was among the 25 to 39-year-olds and cannabis was the most used substance, with an average initiation age of 19 years; amphetamines and ecstasy use among young people was also reported [1]. Prescription opioids, mostly tramadol, morphine, and codeine, were also in high use; others included alcohol and tobacco use [8].

A higher prevalence of substance use, ranging from 20% to 68%, has also been found in different universities in Kenya [9,10].

A study at one Kenyan public university reported a substance use prevalence of 25.5%, with alcohol, cannabis, and tobacco being the most used substances [11].

Research shows that the transition periods from one life event to another are high potential entry points for youths to experiment with substance use and risky behaviours [12]. Students, aged between 18 to 25 years are at the transition point from high school education to college education [13]. This transition is associated with an increased risk for substance use initiation [14]. This risk in Kenya is exacerbated by the long waiting period students take to gain admission into the universities. During this waiting period, idleness may lead the youngsters to start experimenting with alcohol and substance use, behaviours which they may carry with them to the university. Furthermore, many of these students joining the university experience a new freedom from parental and teacher supervision. They additionally become responsible for larger sums of money than ever before in their lives [15]. The combination of these factors increases the susceptibility of the new students to harmful peer influence which may lead to alcohol and substance use initiation.

Age of onset of alcohol and substance use has dropped significantly worldwide from mean age 21 years in the mid-1980s to 10 years in 2012 [16–20]. The ages of 13 to 15 years are the critical years for the onset of substance use in Kenya [21]. Early initiation of substance use is positively predictive of the development of harmful immediate and long term consequences to the users [22]. Young people are particularly vulnerable to the harmful physiological effects of alcohol and substance use because of their immature body systems [20,23]. Psychologically, alcohol and substance use leads to disinhibition and a propensity for risky behaviours among young people [24]. This increases the risk of accidents and injuries, criminal behaviour, poor social relations, sexual assault, and risky sexual behaviour. In the long term, there is an increased risk of poor academic performance and the development of substance use disorders (SUD) [25–27].

Students who take alcohol and substances have been reported, more than their non-drug-using peers, to take longer to complete their studies, they get into trouble with university administration and some get expelled from the universities [28].

Universities, therefore, have to invest resources in the prevention and management of individuals with potential alcohol and substance use disorders to minimize the impact on their academic functioning and psychological wellbeing during their college years.

Programs for the prevention of alcohol and substances abuse are integral to many institutions of higher learning [29]. The strategies used include those aimed at universal prevention for those not yet using, selective prevention for those already experimenting with substances, and treatment of alcohol and substance use disorders for those suffering from harmful substance use [30]. The goal for universal prevention is to prevent young people from initiating substance use, while selective prevention is aimed at those at risk of problematic substance use [30]. In line with these practices, the University of Nairobi has a department on alcohol and substance abuse prevention that carries out activities to educate students on the negative effects of alcohol and substance use. There are also university counsellors who identify those students who abuse alcohol and substances and undertake counselling and rehabilitation [31]. Although the University has a policy of prohibiting alcohol or substance use in all its premises [31], students still get access to and use alcohol and other substances while at the university.

This study, therefore, aimed to determine the prevalence and patterns of alcohol and substance use of the students joining the University of Nairobi. The study findings will help guide universities to design and implement appropriate interventions for the prevention and management of alcohol and substance use among students.

## Methods

### Ethics and consent

The protocol was reviewed and approved by the Kenyatta National Hospital and the University of Nairobi Ethical Committee (KNH-UoN ERC) P98/02/2018. Written informed consent was obtained from all study participants.

### Study area

This study was conducted at the University of Nairobi, which has 61,000 graduate and postgraduate students. Students are either publicly or privately sponsored. They may reside either in on or off-campus residences. The University of Nairobi has seven campuses located in Nairobi city and its environs (www.uonbi.ac.ke). The Chiromo and Kikuyu campuses were purposely selected as the study campuses. This choice was informed by previous surveys that have shown that students on these campuses have a high prevalence of substance use [32]. They were thus selected as the campuses that would provide the determination of the need for and design of prevention and intervention services. The selected campuses also have students who study varied courses in the sciences and humanities which were important to give a full representation of the study domains available in the university education system.

### Study design and population

A cross-sectional study was done on first-year students of the academic year 2018/19 who joined the Kikuyu and Chiromo campuses of the University of Nairobi.

The students in the Chiromo campus take science-based programs like analytical chemistry, astronomy and astrophysics as well as environmental conservation and natural resources management.

Students in the Kikuyu campus take education-based courses like education science, physical education and education arts. These are all 4-year degree programs.

### Sample size calculation

Using the Cochran’s formula for sample size calculation [33], a sample size of 406 respondents was obtained after adjusting for an anticipated 5% non-response rate. Given that Kikuyu campus had an enrolment of 1021 (45.3%) students and Chiromo campus had enrolled 1232 (54.7%) students, out of the sample size of 406, Chiromo proportionately contributed 222 (54.7%) and Kikuyu campus contributed 184 (45.3%).

### Sampling procedure

Sampling probability to population size (PPS) strategy was employed. At the first stage, purposive sampling of the seven campuses of the University of Nairobi selected Kikuyu and Chiromo campuses. This selection was based on the documented high prevalence rates of alcohol and substance abuse among the students of these campuses [32].

At the second stage, total population sampling was done whereby all the six schools making up the Kikuyu and Chiromo campuses were studied. These schools are Education in Kikuyu, and Physical Sciences, Biological Sciences, Mathematics, Biotechnology and Computing in Chiromo.

**Fig 1:**
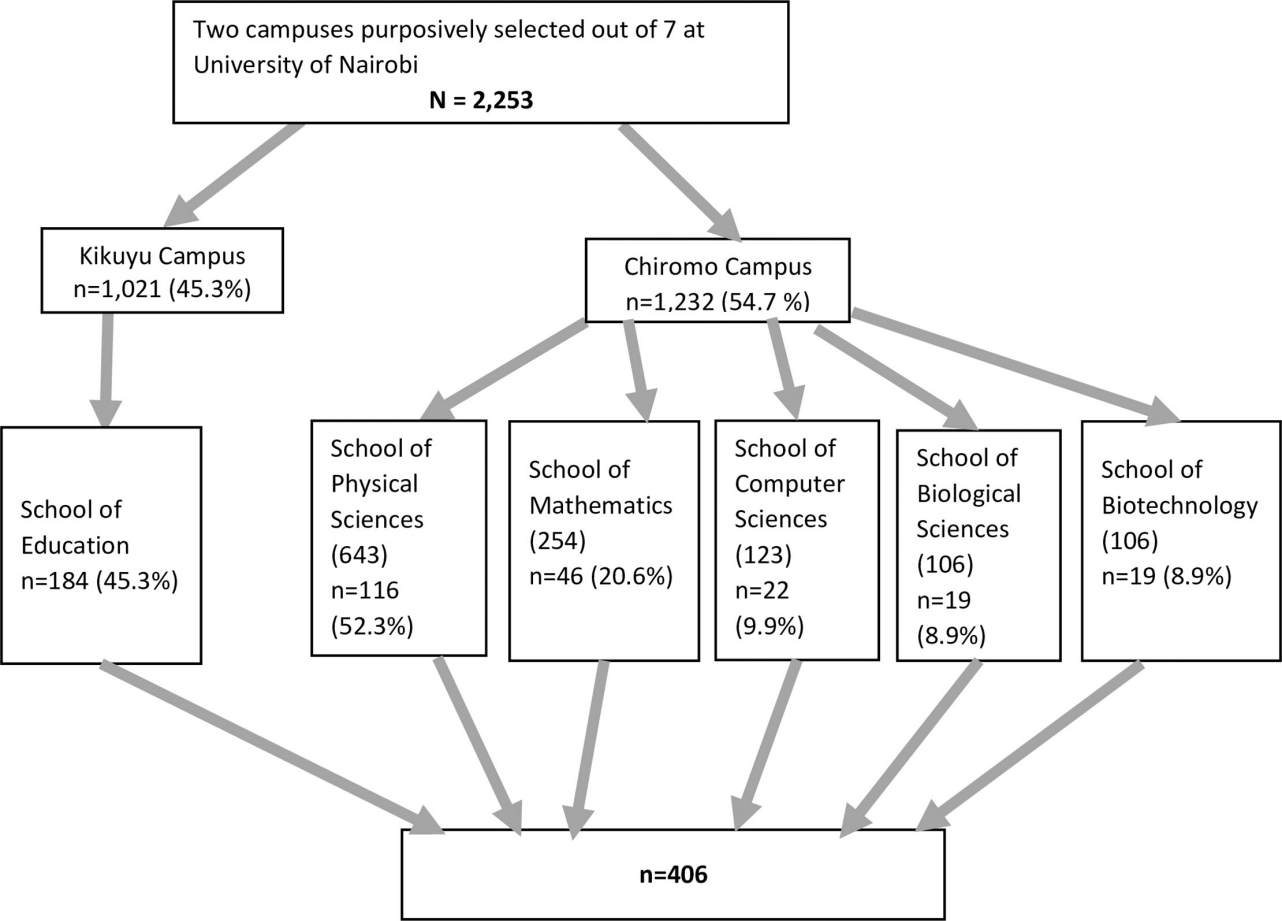
Flow chart of the sampling procedure.

At the third stage, the enrolment lists of first-year students in these schools were obtained and used to make the sampling frame for simple random sampling. The frame comprised 1,021 (45.3%) students in Kikuyu and 1,232 (54.7%) students in Chiromo. Kikuyu Campus with its one school retained the size of its frame for its School of Education. In Chiromo Campus the five schools had each allocated a population-proportionate sampling weight (Fig 1). These various sampling frames were used for the third stage of sampling.

Simple random sampling was applied to each of the constructed sampling frames. First, students were assigned a number starting from 1–1,021 in Kikuyu Campus and 1–1,232 in Chiromo Campus.

Random numbers were generated from the computer software Random.org and used to select study participants from each school. Secondly, the students in each school of Chiromo campus were randomly sampled by application of proportions to the population as summarized in Fig 1. A final total of 406 study participants was selected.

### Data collection

Data were collected between September and November 2018 using the World Health Organization (WHO) Assessment of Smoking and Substance Involvement Test (ASSIST) [34], the Alcohol Use Disorder Identification Tool (AUDIT) [35] and a researcher designed socio-demographic questionnaire.

### Data collection tools

A researcher designed socio-demographic questionnaire was used to collect data on sex, date of birth, age, school of study, course of study, sponsorship (public or private), marital status and residence while studying. The ASSIST tool identifies more than 10 different types of substances including alcohol, cannabis, and tobacco, which are the most commonly used drugs by students [34,36]. Participants were asked if they had ever (lifetime) used alcohol and/or any of the listed substances and if the answer was affirmative, more detailed information was obtained about the previous 3-month frequency and consequences of use of the endorsed substances. The AUDIT-10 tool includes 10 questions that assess alcohol consumption and alcohol-related problems. Response options range from 0 to 4 and a summed total range from 0 to 40 scores. A score of 8 or more indicates hazardous or harmful or probable dependent drinking [37].

The consumption-related items assess binge drinking, defined by the National Institute of Alcohol Abuse and Alcoholism (NIAAA) as the use of four or more standard drinks for women and seven or more standard drinks for men on any one occasion and at least once a week [38]. These tools have been validated for use in various settings involving a wide range of populations [36]. The validity and reliability of the ASSIST and AUDIT tools have been reported as good; they have been adapted for use in Kenya to investigate substance use among university students [10,39].

### Questionnaire administration and retrieval

The questionnaires were administered in classrooms, 30 minutes before a lecture, with permission from the lecturer and assistance of the class representatives. All the students enrolled in a particular school were assigned a unique student number. These numbers were used to make a list. The randomization program (Random.org) was used to generate a list of those numbers to be selected. The students whose numbers were selected were then approached and requested consent to participate in the survey. If the student was not present in class on the day of data collection or if they declined to participate, the student whose number was next on the list was approached and requested to participate. This process was repeated until the required sample size of 406 was achieved. Filled questionnaires were collected by the principal investigator (PI) or research assistants and immediately checked for completeness. They were then transported and securely stored in a locked drawer, which accessible only to the PI to be retrieved later for further cleaning and data processing.

### Variable measures

The main outcomes of alcohol and substance use were assessed as ‘Ever Used’ and ‘Current Use’. Following guidelines for the ASSIST, ‘current use’ was defined by consumption or use of alcohol or other substances in the immediate 3 months preceding the day of data collection. Patterns of alcohol use among the students were measured by the AUDIT-10.

### Data management and statistical analysis

Data were coded and entered using the EpiData 3.1 software. It was checked for inconsistencies and missing values. Incomplete questionnaires were dropped at this point. The cleaned data were exported to Stata software. Data were stratified by study campus and school. The sampling scheme was self-weighing. All statistical analyses were performed using Stata software version 14.2 Special Edition. Stata survey suite was used to adjust for the stratification on data analysis.

Related questions were aggregated to indicate the prevalence of any alcohol or substance used in a lifetime and current use which was defined as use within the immediate 3 months before the day of data collection. Summaries of the lifetime and current use prevalence and social demographic variables were done using descriptive statistics such as mean and mode. Associations between the outcome variables for the lifetime and current use and the independent variables were examined by calculating odds ratios. The variables that were statistically significant at the p < 0.05 levels in bivariate analyses were used to create multivariate models. Multivariate logistical regression was used to assess the impact of explanatory variables on the outcome of the lifetime and current substance use prevalence, for women and men separately.

## Results

### Baseline characteristics of the study respondents

A total of 406 study respondents consented to participate in the study. Table 1 presents the socio-demographic characteristics of the participants. Just over half (222/406, 54.7%) the respondents were registered for courses in Chiromo campus. By sex, approximately half of the respondents 206/406 (50.7%) were male.

**Table 1:** Social demographic characteristics of the study respondents.

Variable		Frequency n = 406	Percentage (%)
**Sex**			
	Male	206	50.7
	Female	200	49.3
**Age (years)**			
	19 and below	264	65
	20 and above	142	35
**School enrolled**			
	Education	184	45.3
	Physical Science	116	28.6
	Mathematics	46	11.3
	Computing	22	5.4
	Biotechnology	19	4.7
	Biological sciences	19	4.7
**Mode of sponsorship**			
	Public-sponsored	371	91.4
	Self-sponsored	35	8.6
**Marital Status**			
	Single	398	98.0
	In a relationship	8	2.0
**Residence**			
	Campus hostels	318	78.5
	Home	62	15.3
	Private hostels	25	6.2

The mean age of all respondents was 19.3± 1.2 years. The majority (371/406 (93.7%), were public sponsored and 318/406 (78.5%) resided on campus at university hostels.

### Prevalence of substance use among the study respondents

The prevalence rates of substance use among the study respondents are as presented in (Table 2). Overall 103 respondents (25.4%, 95% CI:21.21, 29.90) had ever used alcohol or another substance in their lifetime. Alcohol was the most used substance, having been ever used by 89/406, (21.9%, 95% CI:17.99, 26.27) of the study respondents in their lifetime. Cannabis was ever consumed by 33/406 (8.1%, 95% CI: 5.66, 11.23) of the study respondents. All the other groups of drugs listed (opioids, cocaine, amphetamines, hallucinogens, sedatives, and inhalants) had been used by (9.4%, 95% CI: 6.71, 12.62) of the respondents. Males had a higher prevalence of lifetime substance use at 63/206 (30.6%, 95% CI: 24.37, 37.36) compared to females at 40/200 (19%, 95% CI:13.81,25.13). This pattern was replicated across most substances assessed.

**Table 2:** Prevalence and associated confidence intervals by sex among the study respondents.

**Substance**	**Male**	**Female**	**Overall**
	**Ever use in lifetime**		
Alcohol	27.67 (21.68, 34.31)	16.00 (11.21,21.83)	21.92 (17.99, 26.27)
Tobacco	10.19 (6.4, 15.61)	3.50 (1.42, 7.08)	6.90 (4.63, 9.81)
Cannabis	12.62 (8.4, 17.94)	3.50 (1.42, 7.08)	8.13 (5.66, 11.23)
Others*	9.22 (5.64, 14.02)	9.50 (5.80, 14.44)	9.36 (6.71, 12.62)
Overall	30.58 (24.37, 37.36)	19.00 (13.81,25.13)	25.37 (21.21, 29.90)
	**Current use (use in last 3 months before study)**		
Alcohol	22.81 (17.27, 29.16)	11.00 (7.02, 16.18)	16.99 (13.47,21.01)
Tobacco	7.77 (4.50, 12.31)	2.50 (0.82, 5.74)	5.17 (3.23, 7.80)
Cannabis	8.25 (4.88, 12.88)	2.00 (0.55, 5.04)	6.90 (4.63, 9.81)
Others*	9.22 (5.64, 14.03)	9.50 (5.82, 14.44)	9.36 (6.71, 12.62)
Overall	26.21 (20.35, 32.78)	14.50 (9.93, 20.16)	20.44 (16.62, 24.70)

*opioids, cocaine, amphetamines, hallucinogens, sedatives, and inhalants.

The overall prevalence of recent substance use (reported use in the last three months) was 83/406 (20.4%, 95% CI: 16.62, 24.70). A similar pattern to that of ever use of substances was reported, with males’ current use of alcohol (22.8%,95% CI:17.27,29.16) being double the females’ rates of use for alcohol (11.00% 95% CI: 7.02, 16.18), this higher pattern of substance use among the males was replicated across most of the other substances (Table 2).

### Patterns of current substance use (use within last 3 months before the study)

The study participants who reported current alcohol use 42/406 (10.3%) drank twice or more times per month, 15/406 (3.7%) drank at least once a month while 8/406 (2%) drank alcohol every week and 4/406 (1%) drank daily (Table 3). Weekly use of cannabis was reported by 6/406 (1.5%) of the users, while 13/406 (3.2%) used cannabis twice or more times per month. Out of the six respondents who reported use of cocaine two reported daily use, while the others used at least once a month (Table 3).

**Table 3:** Patterns of substance use among study respondents within the last 3 months.

Substance	Frequency of Current use (within the last 3 months)				
	Never	Twice or more a month	Monthly	Weekly	Daily
	Frequency (%)	Frequency (%)	Frequency (%)	Frequency (%)	Frequency (%)
Alcohol	337 (83)	42 (10.3)	15 (3.7)	8 (2)	4 (1)
Tobacco	383 (94.3)	14 (3.5)	3 (0.7)	1 (0.2)	3 (0.7)
Cannabis	385 (94.8)	13 (3.2)	1 (0.2)	6 (1.5)	1 (0.2)
Sedatives	392 (96.6)	10 (2.5)	3 (0.7)	0 (0)	1 (0.2)
Hallucinogens	396 (97.5)	8 (2)	1 (0.2)	1 (0.2)	0 (0)
Inhalants	401 (98.8)	5 (1.2)	0 (0)	0 (0)	0 (0)
Amphetamine	401 (98.8)	4 (1)	1 (0.2)	0 (0)	0 (0)
Cocaine	400 (98.5)	2 (0.5)	2 (0.5)	0 (0)	2 (0.5)
Opioids	404 (99.5)	1 (0.2)	1 (0.2)	0 (0)	0 (0)

### Predictors of current and lifetime substance use among study respondents

Female first-year students had a significantly lower odds of current substance use compared to their male counterparts, (Odd Ratio (OR) 0.43(0.19–0.95), p<0.005) (Table 4).

**Table 4:** Predictors of the lifetime and current substance use among study respondents adjusting for sampling weights.

	Lifetime use				Current use			
Variable	Odds Ratio	P-value	[95% Confidence interval]		Odds Ratio	P-value	[95% Confidence interval]	
**Sex**								
Male	**1**				**1**			
Female	0.55	0.12	0.26	1.16	0.43	0.04*	0.19	0.95
**Age category**								
19 and below	**1**				**1**			
20 to 25	1.99	0.05*	0.985	4.024	1.38	0.41	0.65	2.93
**Sponsorship**								
Public-sponsored	**1**				**1**			
Self-sponsored	1.19	0.75	0.40	3.56	1.81	0.31	0.58	5.68
**Residence**								
Campus Hostels	**1**				**1**			
Private Hostels	4.27	0.02*	1.25	14.62	4.40	0.03*	1.15	16.86
Home	1.29	0.65	0.44	3.75	1.47	0.5	0.48	4.53
**School enrolled**								
Physical sciences	**1**				**1**			
Mathematics	0.74	0.52	0.29	1.88	1.28	0.61	0.48	3.41
Biological science	3.15	0.04*	1.05	9.51	5.12	0.006*	1.60	16.38
Computing	1.95	0.23	0.65	5.85	1.76	0.31	0.59	5.23
Education	0.89	0.72	0.48	1.66	1.28	0.49	0.64	2.56
Biotechnology	2.37	0.19	0.65	8.59	2.63	0.16	0.68	10.26

*Significant at p≤0.05.

There was no significant difference in odds of current substance use between students who were government-sponsored compared to the privately sponsored ones. However, students who lived in private hostels were four times more likely to be current substance users as compared to those who resided in campus hostels (OR 4.40(1.14–16.86), p<0.005) (Table 4).

The odds of current substance use by respondents from the School of Biological Sciences were five times those of the respondents from the School of Physical sciences (OR 5.10 (1.60–16.38), p<0.05) (Table 4).

## Discussion

This study examined the prevalence, patterns, and predictors of the lifetime and current alcohol and substance use among first-year students at the University of Nairobi.

The overall lifetime substance use prevalence was found to be 25.4%. This is a considerably high rate, with nearly a quarter of the respondents using alcohol and other substances at admission to the university. The findings of this study are comparable with those of a similar study among students of Kenyatta University which found a lifetime substance use prevalence of 25.1% [11]. The comparability of findings may be due to students of the two universities being drawn from similar catchment populations as well as staying in the same urban settings.

The study findings are however, lower than those found in a study done among college students in Eldoret, Kenya, which found a lifetime substance use prevalence of 69.7% [9]. Given that the Eldoret study and the present one had respondents of similar age and education, differences in geographical locations and settings between Eldoret and Nairobi may explain the disparities of findings. Eldoret is located in a more rural setting as compared to the very metropolitan Nairobi, the capital city of Kenya. It is expected that students in the major cities have more vulnerabilities as well as opportunities for alcohol and substance use. Nevertheless, students in a major city may be more exposed to information on the negative effects of substance use due to connection to the internet and programs that target the prevention of substance use among university students. Moreover, the study done in Eldoret was done eight years earlier; a lot has changed in Kenya in terms of legislation concerning alcohol and substance use, and implementation of the Alcoholic Drinks Control Act 2010, revised 2012 [40].

The national prevalence of lifetime substance use in Kenya, for those aged 15–24 years, is 37.1% while the current substance use is 19.8% [19], these figures are higher than the lifetime substance use prevalence of 25.4% and 17% current substance use found by this study. However, this study found a higher prevalence of current alcohol and cannabis use at 17% and 5.2% compared to the Kenyan national prevalence of 11.7% and 1.5% respectively [19]. These higher trends of the current use of alcohol and cannabis by university students may be explained by the normalizing of substance use behaviour and aggressive marketing of alcohol and other substances among college students through digital platforms and social media like Facebook and WhatsApp, of which college students are prolific users [41].

The prevalence of substance use in studies across different geographical locations and social-economic environments in Africa showed comparable results to those reported by our study. In Northern Tanzania, a study by *Francis et al* reported a prevalence of current alcohol use of 45% among male college students and 26% among female students [42]. In South Africa, *Ramsoomar et el*, in a study done among adolescents at 13 and 18 years, found that the prevalence of lifetime use of substances rose from 22% at 13 years to 66% at 18 years [18]. A study among medical students in Nigeria reported a lifetime prevalence of use of mild stimulants of 46.1%, alcohol, 39.7%, and tobacco, 6% [43]. The prevalence of stimulant use in the Nigerian study was higher than the prevalence reported in our study. Besides, the use of alcohol is more common among our study respondents as compared to the use of stimulants. However, the prevalence rates of tobacco and cannabis were comparable to the findings in our study. This may be explained by the fact that all the study sites are in urban settings and the risks and exposures to substance use are comparable in most of the cities.

Prevalence studies done in European countries showed some similarities as well as contrasting findings. In France, a study among university students reported the prevalence of alcohol and tobacco consumption was at 20.1% and 23.2% respectively [44]. Studies among university students in nine member countries of the Association of South-East Asian Nations (ASEAN) found varying prevalence rates of illicit drugs, ranging from 0.2% in Cambodia to 45.7% in Laos [26]. The prevalence rates of our study fall within these ranges.

In the study among French students, even though the prevalence of alcohol use was comparable to the findings of our study, the reported 23.2% prevalence of tobacco consumption was higher than the 5.1% current use reported by our study. This difference in tobacco use among French study respondents and the respondents in our study may be accounted for by the tobacco control measures by the government of Kenya through the enacted Tobacco control act 2007 revised (2012), which has prohibited tobacco use in all public places among other controls [45]. These differences also support the premise that government legislation and enforcement are important in controlling substance use among young people.

The results for the prevalence of lifetime substance use by age showed a slightly higher rate among students who were 20 years and above compared to those aged below 19 years. There was, however, little difference in the prevalence of current substance use among students in the two age categories. Studies have shown that substance use behaviour is highest at the age of 18 to 24 years [46]. Young people at this age pursue their university education; at the same time, the age of initiation to alcohol and substance use has reportedly reduced in recent years [20]. In Kenyan studies, university students are among the leading categories of substance users [10].

There was a sex difference in the use of substances; males had higher rates of use in all categories of substances. These results are similar to those from other parts of the world in which males college students have been found to have higher rates of substance use than females students [5,42,47]. This trend may be a result of societies that are more tolerant of substance use among men as opposed to women.

The residence of the university students was associated with differences in the rates of substance use. The study results indicated that 48% of the students who reported current alcohol and substances use resided in private hostels.

Those who resided at home with parents were the second-largest users, while those who resided in university hostels reported the least substance use. These findings contradict an earlier study by *Simons-Morton et*. *al*, which found that students who resided in campus residences had the highest substance use [48]. The findings, however, are in agreement with a study that found that living in off-campus residences posed a high risk of alcohol use [49]. Private student hostels in Kenya do not have strict enforcement of rules regarding the use of alcohol and drugs in their premises. The landlords would not want to lose tenants by enforcing restriction measures on substance use by the student residents. This therefore, accords the resident students liberties to behave as they please, thus making them more vulnerable to substance use. This phenomenon could also be as a result of the difference in the social-economic status of students who reside in on or off-campus accommodation. In Kenya, students who reside in off-campus residences are often from a higher social economic background as compared to those using on-campus accommodation facilities. This underpins the need to have prevention interventions among university students target both on-campus and off-campus residents. Most of the preventive interventions for alcohol and substance use among university students focus on-campus facilities, this leaves out a vulnerable group in off-campus student residents.

The patterns of alcohol use found in the study also reveal that most students only used alcohol occasionally. This was most likely during the weekends when they socialize with their friends. This is of concern because these students often may take many substances at one sitting, as well as mixing different types of substances. Research evidence shows that students who binge drink are most likely to suffer acute and negative consequences of substance use [46]. There were 5% of students who reported regular use of alcohol, some even daily use. These students are of concern given that they are at great risk of having their substance use alter the cognitive and physical functioning. This potentially would lead to poor academic outcomes as well as violence and criminal activities as has been documented in previous studies [23,50].

Given the high number of students who used alcohol and other substances by the time they reported for university education, preventive interventions should start at the pre-university level. These levels include at high schools and home environments and they should be intensified throughout their university education period.

Current models of delivering alcohol and substance use prevention interventions include face-to-face interventions with the college students however, these are difficult to implement, because of the stigma associated with substance use. As a result, only 10–15% of college students receive the interventions needed to address their substance use problems [51]. Innovative and youth-friendly intervention strategies are key to prevent alcohol and substance use among university students [30]. The use of technology-based interventions would provide a more acceptable avenue to deliver evidence-based interventions to college students compared to face-to-face programs [30]. University management should explore all avenues to provide innovative strategies for the prevention of alcohol and substance use, as well as related negative consequences, among their students.

### Strengths of the study

This study provides epidemiological information about the prevalence and patterns of alcohol and substance use among first-year university students. Furthermore, the results of this study have positive implications for strengthening interventions on substance use prevention among university students as well as a reference point for future comparative studies.

### Limitations of the study

This study used a cross-sectional design and data; as such, this precluded any causal associations to be made on the factors associated with alcohol and substance use.

Also, the self-report measures employed in data collection may have led to recall and reporting bias as students may have given socially desirable responses, thus potentially leading to over/under-reporting of the prevalence of substance use.

To minimise on recall bias, we used pictorial charts of standard alcoholic drinks to aid the respondents. Moreover, we emphasized to the respondents the need to be truthful as their responses were confidential. This study was done on two campuses of a single public university; thus the findings may not be generalizable to other universities.

## Conclusions and recommendations

This study demonstrated that the prevalence of alcohol and substance use among first-year university students at the Kikuyu and Chiromo campuses of the University of Nairobi is high. Interventions for the prevention and management of alcohol and substance use should therefore, start as early as at the entry to university. Thematic orientation programs are key to educate first-year university students on the negative effects of alcohol and substance use. Life skills training should be instituted to help students adjust and adapt to their university life well. Off-campus residence, advanced age, and male gender of university students were found to be positively predictive of their use of alcohol and other substances. Therefore, alcohol and substance use prevention intervention strategies should allow extra focus on these vulnerable sub-groups. Universities should also increase the available accommodation facilities for their students since living in on-campus residences was found to be associated with lower rates of substance use. This would also make prevention interventions among college students easier to implement.

We propose that programs for alcohol and substance use prevention and education should have a multi-sectoral approach, which should start at high school level and be intensified as the young adults join university education.

## Supporting information

S1 Data Alcohol substance use data PLOSONE. (XLSX)
